# Hypertension and Device-Based Therapies for Resistant Hypertension: An Up-to-Date Review

**DOI:** 10.7759/cureus.66304

**Published:** 2024-08-06

**Authors:** Oluwaremilekun Tolu-Akinnawo, David N Ray, Tiwalade Awosanya, Chike Nzerue, Henry Okafor

**Affiliations:** 1 Internal Medicine, Meharry Medical College, Nashville, USA; 2 Medicine, Meharry Medical College, Nashville, USA; 3 Endocrinology, Meharry Medical College, Nashville, USA; 4 Cardiology, Vanderbilt University Medical Center, Nashville, USA

**Keywords:** cardiovascular disease (cvd), renal denervation therapy, device-based therapy, treatment-resistant hypertension, resistant hypertension

## Abstract

Hypertension is the most prevalent modifiable risk factor associated with cardiovascular mortality. The World Health Organization (WHO) estimates that hypertension directly or indirectly causes the death of at least nine million people globally every year. The number of people living with hypertension (blood pressure (BP) of ≥140 mmHg systolic or ≥90 mmHg diastolic or on medication) doubled between 1990 and 2019, from 650 million to 1.3 billion. Despite a plethora of antihypertensive drugs widely available, a sizable part of the antihypertensive population stays uncontrolled. The unmet need of controlling BP in this population may be addressed, in part, by developing new drugs and devices/procedures to treat hypertension and its comorbidities. Several device-based approaches have been introduced to lower BP, and most of these strategies aim to modulate autonomic nervous system activity. Importantly, when considering a device-based treatment, each patient's underlying pathophysiology is considered, and the procedural risks are weighed against the cardiovascular risk attributed to the elevated BP. In November 2023, the FDA approved two renal denervation (RDN) devices. This manuscript discusses current interventional devices and procedures recently approved (RDN) and others in the clinical testing stage for arterial hypertension intervention or management. As we list below, all others have shown promising results and are being evaluated on a larger clinical trial. The new device-based classes are as follows: catheter-based RDN, baroreflex amplification, arteriovenous (AV) malformation, carotid body (CB) ablation, pacemaker-based cardiac neuromodulation, electro-acupuncture, and deep brain stimulation. Baroreflex amplification uses peripheral neuromodulation, while AV malformation leverages AV anastomosis. CB ablation modulates chemoreceptors, and pacemaker-based neuromodulation adjusts atrioventricular intervals. Electro-acupuncture proves potential, and deep brain stimulation offers central nervous system intervention.

## Introduction and background

WHO classifies hypertension as “the silent killer” and describes us as being in a race against it. This is because patients could remain asymptomatic prior to the onset of complications. Hypertension and its associated complications also have enormous economic costs - for patients and their families, health systems, and national economies. People living with the condition incur direct medical costs and lost wages, often in their prime working years, which can be impoverishing for entire families [1]. Hypertension remains a critical global health concern and a significant contributor to morbidity and mortality globally. According to the WHO, the burden of untreated hypertension continues to be on the rise, affecting over one billion individuals worldwide and leading to potentially life-threatening cardiovascular complications [2]. This worsening crisis has led to ongoing developments in treatment strategies, mainly focusing on poorly controlled hypertension. 

The 2017 guidelines from the American College of Cardiology (ACC) and American Heart Association (AHA) redefined hypertension as an elevated systolic blood pressure (SBP) of 130 mmHg or higher or diastolic blood pressure (DBP) of 80 mmHg or higher. This is a more stringent criterion compared to the 2003 guidelines, resulting in an increased overall prevalence of hypertension from approximately 32%-47% [3,4]. Individualized treatment goals are emphasized, considering various co-morbidities and tailoring blood pressure targets to the patient's characteristics, preferences, and tolerance. For instance, specific objectives are outlined for patients with cardiovascular disease, chronic kidney disease (CKD), and diabetes, reflecting recommendations from the 2021 Kidney Disease Improving Global Outcomes guidelines and the American Diabetes Association [5-7].

The ACC-AHA defines resistant hypertension (RH) as poorly controlled blood pressure despite the patient receiving at least three medications with different mechanisms of action, including a diuretic, even at maximally tolerated doses [8]. RH is also categorized as controlled blood pressure on at least four medications with different mechanisms of action. The term "pseudo-resistant hypertension" is introduced to describe poorly controlled blood pressure resulting from poor measurement technique, medication non-compliance, or white-coat hypertension [8]. This is important as in the case of pseudo-RH, targeting the factors responsible reverse the poorly controlled hypertension. 

Determining the prevalence of RH is challenging due to the necessity to rule out pseudo-RH. However, estimates from the National Health and Nutrition Examination Survey (NHANES) suggest a prevalence of approximately 12.8% in the general population, using a cutoff of BP >/= 140/90 mmHg, and 40.4% in CKD [9,10]. Anticipated factors contributing to an increase in this percentage include an aging population, rising obesity rates, and sedentary lifestyles all through different mechanisms.

RH is associated with adverse outcomes, including kidney failure, cardiovascular morbidity, and death [11-13]. A recent study involving 10,001 patients with apparent treatment - RH - revealed a 64% higher incidence of composite cardiovascular complications, such as fatal coronary heart disease, nonfatal myocardial infarction, cardiac arrest, and cerebrovascular accidents [13]. Confirmation of true RH necessitates optimizing the drug regimen following recommendations from the European Society of Cardiology (ESC) and AHA, incorporating lifestyle modifications and meticulous drug management to enhance blood pressure control [14]. Persistent poor blood pressure control prompts the search for secondary causes of hypertension, and interventional procedures become a consideration.

Refractory hypertension, as defined by the AHA, denotes severe uncontrolled hypertension despite being on five antihypertensive medications from different classes, including mineralocorticoids and thiazide diuretics [15]. This has spurred the resurgence of interest in device-based therapies, seen as an adjunct to medical treatment. The revitalization of these therapies addresses the need for better hypertension control, particularly in cases of non-adherence to drugs, emphasizing the inhibition of the renin-angiotensin-aldosterone system. After extensive experimental and clinical research over the past two decades, the scientific community has better understood the pathophysiology responsible for reducing blood pressure. As of November 2023, two procedural options for treating resistant/refractory hypertension, namely, ultrasound renal denervation (RDN) and radiofrequency RDN, have received FDA approval.

This article provides an in-depth and up-to-date review of device-based therapies for RH, integrating critical insights from guidelines, studies, and developments in the field.

## Review

Device-based therapies

RDN

RDN is currently the most developed and established therapy for RH. Accounting for nearly 70% of RH therapy coverage, more than 10 RDN systems are clinically approved [16]. Despite being device-based, RDN is not a permanent implant but rather requires only a single surgical intervention.

Pathophysiology of RDN: The efficacy of RDN revolves around the modulation of renal nerves, which play a critical role in kidney function and blood pressure (BP) control. These nerves, particularly the efferent nerves, significantly influence renin release, sodium retention, vasoconstriction, and overall BP regulation [17]. This understanding has driven extensive human and animal trials to assess the safety and effectiveness of RDN.

Clinical trial overview:

(1) SYMPLICITY trials. The SYMPLICITY HTN-1 and SYMPLICITY HTN-2 trials, initiated in 2009, were the first to demonstrate promising results in BP control for patients with RH. However, the subsequent SYMPLICITY HTN-3 sham-controlled trial yielded less favorable results, showing no significant superiority of RDN over the sham procedure [18-20]. These outcomes prompted the development of the DENER HTN trial, which achieved positive results by addressing issues related to patient selection and procedural execution identified as potential causes for the SYMPLICITY HTN-3 trial's failure [21,22].

(2) SPYRAL HTN and RADIANCE-HTN trials. The SPYRAL HTN and RADIANCE-HTN trials focused on multi-electrode radiofrequency (RF) denervation and ultrasound (US) denervation, respectively. Both trials demonstrated the superiority of these methods over the sham procedures [23-27]. Additionally, research into alcohol-mediated denervation and cryo-RDN is gaining momentum, showing promising future applications for these techniques [28-31].

Mechanism of action: Catheter-based RDN alters sympathetic renal activity by disrupting afferent and efferent nerve fibers in the renal artery adventitia via a minimally invasive procedure [32,33]. The RDN catheters employ thermal or chemical ablation methods to interrupt renal sympathetic nerve signaling. US and RF-based catheters achieve this through thermal ablation, while alcohol denervation utilizes chemical ablation delivered via a three-needle device [34,35].

Although pharmacological therapy has proven effective in reducing the risk of coronary heart disease, stroke, and heart failure, it remains unclear whether RDN can provide the same benefits. Further research is needed to establish this, despite observational studies and meta-analyses suggesting positive effects on target organ damage [36,37].

Challenges to implementation:

(1) Procedural and technical challenges. One of the significant challenges for integrating RDN into clinical practice is related to operator skill and experience. A successful RDN implementation requires highly skilled operators to perform the procedure accurately and safely. Variability in operator experience can impact outcomes, necessitating extensive training and certification programs. Another potential barrier is the standardization of techniques. There is a lack of standardization in RDN techniques and protocols, leading to variations in procedural success and patient outcomes. Establishing standardized guidelines is critical to providing consistent results.

(2) Device and technology limitations as a potential challenge. As the development and refinement of RDN devices continue to evolve, differences in device efficacy, safety profiles, and costs need to be addressed to optimize the use of RDN.

(3) Clinical challenges. Clinically, patient selection is a challenge. Identifying appropriate candidates for RDN is crucial. Factors such as comorbid conditions, anatomy of renal arteries, and baseline BP levels influence the selection process. Misidentification can lead to suboptimal outcomes. This is particularly one of the most challenging aspects of RDN implementation. Another barrier to the implementation of RDN is the data on long-term efficacy and safety. Long-term data on the effectiveness and safety of RDN are still limited. Ongoing studies are required to confirm sustained BP reductions and assess potential long-term adverse effects.

(4) Regulatory and economic challenges. Due to concerns regarding safety and efficacy, RDN devices must undergo rigorous regulatory evaluations to ensure safety and efficacy. The approval process can be lengthy and varies by region, potentially delaying access to new technologies. As of November 2023, US and RF RDN have gained FDA approval.

Cost and reimbursement also continue to be significant barriers to RDN implementation. The cost of RDN procedures and devices can be high. Ensuring adequate reimbursement from healthcare systems and insurance providers is essential to make the therapy accessible to patients. This means less likely access to the underserved population, further bridging health disparities.

The availability of required healthcare infrastructure is another potential barrier. This is because the implementation of RDN requires well-equipped healthcare facilities with advanced imaging and procedural capabilities. Infrastructure limitations in certain regions may hinder widespread adoption, especially in underserved/low-resource settings.

Recent advances in RDN:

(1) US RDN. The introduction of a non-invasive US RDN therapy presents a more appealing method for renal nerve ablation. This technique uses externally focused energy with a diagnostic Doppler device for precise targeting and tracking. Initially developed by Kona Medical, Inc. in California, at least six trials have validated its BP-lowering efficacy in humans [38]. However, a recent sham-controlled trial did not show significant differences between the sham and US RDN groups. However, a greater ambulatory BP (ABP) change was noted in the US RDN group due to BP stabilization at baseline [39]. The surround sound system, which uses externally delivered ultrasound to achieve RDN, appears to be a promising approach to RDN by eliminating several of the factors currently limiting the intravenous approach, as it is less invasive and likely to be more accepted [38].

(2) Alcohol-mediated RDN. A recent trial involving 45 patients with uncontrolled hypertension on multiple medications showed that a bilateral infusion of 0.6 mL of alcohol per artery resulted in significant BP reductions. Ambulatory BP decreased by 11/7 mmHg (95% CI: -15 to -7 or -9 to -4 mmHg) and office BP by 18/10 mmHg (95% CI: -25 to -12 or -13 to -6 mmHg) at six months [28]. The procedure was relatively short and exhibited a favorable safety profile. Two larger randomized, sham-controlled trials, TARGET BP OFF-MED and TARGET BP 1, are underway to investigate these findings further [40].

The catheter-based RDN therapy continues to evolve as a promising therapy for RH, driven by technological advancements and clinical research. While challenges and uncertainties remain, ongoing trials and studies aim to refine the techniques and validate their long-term efficacy and safety. Addressing the procedural, clinical, regulatory, and economic challenges will be crucial for the widespread adoption and success of RDN. As research progresses, RDN has the potential to impact the management of hypertension and improve patient outcomes significantly.

Baroreflex Amplification

Baroreflex amplification therapy is an innovative approach to managing RH through peripheral neuromodulation. This method leverages the body's natural baroreflex mechanism, where increased BP levels stimulate stretch-sensitive baroreceptors located in the carotid sinus and aortic arch. This stimulation triggers a rapid negative feedback loop that enhances afferent signaling and reduces efferent sympathetic outflow, decreasing heart rate and total peripheral resistance. Consequently, BP returns to an adequate level [40].

Historical context and technological evolution: Baroreceptor stimulation was initially studied in the 19th century, marking the first generation of research in this field. However, early attempts were abandoned due to technological and safety concerns. With advancements in medical technology and the increasing focus on device-based therapies for RH, baroreflex amplification experienced a revival approximately two decades ago.

Clinical trials and developments:

(1) First-generation trials. The Rheos Pivotal trial [41] represented the first generation of baroreflex amplification trials. Despite its pioneering nature, the trial did not achieve Food and Drug Administration (FDA) approval and was consequently discontinued. One of the reasons it was discontinued is its lack of efficacy as the reduction in blood pressure in the treatment group was not significantly better than that of the control group, which received a sham procedure [41]. Additionally, there were some safety concerns including nerve injury, wound infections, device-related complications such as lead dislodgment, and system malfunction [41].

(2) Second-generation trials. A more recent cohort study involving the Barostim Neo device demonstrated long-term efficacy in SBP control, achieving levels below 140 mmHg in 25 out of 50 patients [42] through a multi-center, randomized, controlled clinical trial. However, further randomized controlled clinical trials must validate its effects on ambulatory BP.

Endovascular baroreflex amplification (EVBA): There are ongoing studies to develop less invasive devices that stimulate the baroreflex region, such as the EVBA MobiusHD device. This device is designed to be implanted inside the carotid sinus. Experimental studies comparing a conventional self-expanding stent with the MobiusHD device have shown immediate and sustained BP-lowering effects, with MobiusHD also demonstrating a better safety profile [43].

The CALM-FIM study, which was the first-in-man trial conducted across Europe and USA centers in May 2013, reported significant reductions in-office BP (OBP), both systolic and diastolic, after six months (24 and 12 mmHg, respectively) [43]. After three years, SBP decreased by 30 mmHg, further supporting the efficacy of this approach. However, adverse safety endpoints were recorded in five patients, necessitating immediate interventions for hypotension, worsening hypertension, and infections [43].

Ongoing trials: Current trials, such as CALM-2, a prospective, randomized, double-blind, sham-controlled pivotal study, thoroughly evaluate EVBA with the MobiusHD device [43]. The primary outcome of these studies is the change in mean 24-hour systolic ambulatory BP (ABP) over six months [43].

Challenges to implementation:

(1) Technological challenges. A significant challenge for implementing baroreflex amplification therapy is the device complexity. The complexity of baroreflex amplification devices requires sophisticated technology and precise engineering to ensure efficacy and safety. Continuous innovation and improvement are necessary to enhance device performance and patient outcomes.

Another potential challenge is its long-term efficacy and durability. Ensuring baroreflex amplification devices' long-term effectiveness and durability remains a significant challenge. Devices must maintain their performance over extended periods without causing adverse effects.

(2) Clinical challenges. Clinically, a potential challenge to baroreflex amplification therapy implementation is patient selection, which is crucial. Identifying suitable candidates for baroreflex amplification is vital. Patients must be carefully selected based on their specific medical conditions, anatomy, and response to previous treatments to maximize the benefits and minimize risks.

Managing adverse events such as hypotension, worsening hypertension, and infections is critical. The incidence of such events must be minimized through improved device design and procedural techniques.

(3) Regulatory and economic challenges. Due to concerns regarding its safety and efficacy, baroreflex amplification therapy implementation must undergo stringent regulatory evaluations. The regulatory process can be time-consuming and varies by region, potentially delaying patient access to new treatments.

The high cost of baroreflex amplification devices and procedures poses a challenge. Ensuring adequate reimbursement from healthcare systems and insurance providers is essential to make this therapy accessible to a broader patient population. This poses a significant barrier to implementation in the underserved/low-resource setting, further bridging the gap in health disparities.

Implementing baroreflex amplification requires advanced healthcare facilities with specialized equipment and trained personnel. Infrastructure limitations in certain regions may hinder the widespread adoption of this technology.

Baroreflex amplification represents a promising alternative to RDN for managing resistant hypertension [40]. With ongoing technological advancements and clinical research, this method can potentially benefit BP control significantly. However, addressing the technological, clinical, regulatory, and economic challenges is essential for successfully implementing and adopting baroreflex amplification. Continued research and innovation are critical to overcoming these challenges and improving patient outcomes in managing resistant hypertension.

Arteriovenous (AV) Malformation

The ROX coupler device creates a fixed-diameter (typically 4 mm) AV anastomosis between the external iliac artery and vein. This connection links a low-resistance, high-compliance venous segment to the central arterial tree, immediately reducing blood pressure (BP). The coupling leads to a decrease in systemic vascular resistance and an increase in cardiac output, contributing to the overall reduction in SBP and DBP [44-47].

Clinical trials and evidence: Initial development and COPD treatment. The ROX coupler was initially developed to treat chronic obstructive pulmonary disease (COPD) by increasing mixed venous oxygen saturation. In an open-label study involving 24 patients with COPD, the creation of an AV fistula (AVF) unexpectedly resulted in reduced systemic vascular resistance and increased cardiac output, which led to a notable drop in both SBP and DBP from baseline to the 12-month follow-up [45,48]. This phenomenon is similar to the reduction in BP observed in patients with end-stage renal disease following AVF creation [48]. The reduction in BP could be beneficial in reducing hypertension-associated complications, such as cerebrovascular artery (CVA) disease, and myocardial infarction (MI); however, it could potentiate high-output heart failure. There are also concerns for hemorrhage, infection, device migration, thrombosis, and stenosis.

ROX CONTROL-HTN study: The efficacy of the ROX coupler in treating RH was further demonstrated in the ROX CONTROL-HTN study. This study involved 83 patients with RH and showed a significant mean systolic reduction of 13.5 ± 18.8 mmHg in patients with the AV coupler, compared to a negligible change of 0.5 ± 15.8 mmHg in the control group [48]. One-year follow-up data indicated a sustained reduction in both OBP and ABP. Specifically, OBP showed a decrease of SBP by 25.1 ± 23.3 mmHg and DBP by 20.8 ± 13.3 mmHg (p < 0.0001 for both), while ABP demonstrated a reduction in SBP by 12.6 ± 17.4 mmHg and DBP by 15.3 ± 9.7 mmHg (p < 0.0001) [44]. Additionally, there was a sustained reduction in isolated systolic hypertension [47].

Challenges to implementation:

(1) Technological and procedural challenges. Device placement and technical expertise are potential barriers to implementing AV malformation therapy. This is because the creation of an AV anastomosis requires precise surgical skill and expertise to ensure accurate placement and minimize complications. Misplacement or technical errors can lead to ineffective outcomes or adverse events. Another potential barrier is the long-term durability of the device. Ensuring the long-term functionality and durability of the ROX coupler device is critical. Devices must withstand the physiological stresses over time without degrading or causing complications.

(2) Clinical challenges. Clinically, identifying suitable candidates for the ROX coupler procedure is essential. Patients must be carefully evaluated to ensure that they are appropriate for the device, considering their overall health, comorbid conditions, and specific anatomical factors. Another challenge to the implementation of AV malformation device therapy is the management of potential complications. The potential risk of iliac venous stent complications, such as thrombosis or stenosis, poses a significant clinical challenge. Effective management and prompt intervention are necessary to address these complications and ensure patient safety.

(3) Regulatory and economic challenges. Due to the potential side effects, the ROX coupler device must meet stringent regulatory standards to ensure its safety and efficacy. Obtaining and maintaining regulatory approval across different regions can be complex and time-consuming.

AV malformation device therapy implementation is expensive, and the high cost of the ROX coupler device and associated procedural expenses can be a barrier to widespread adoption. Ensuring adequate reimbursement from healthcare systems and insurance providers is crucial to making this therapy accessible to a broader patient population.

Implementing the ROX coupler procedure requires advanced healthcare facilities equipped with specialized tools and trained personnel. Developing and maintaining such infrastructure can be challenging, especially in resource-limited settings.

Without much doubt, the ROX coupler device offers a promising solution for managing resistant hypertension through its unique mechanism of creating an AV anastomosis. Clinical trials have demonstrated its efficacy in significantly reducing blood pressure [44-47]. However, the successful implementation of this technology faces several challenges, including technological, clinical, regulatory, and economic barriers. Addressing these challenges through ongoing research, improved device design, and robust clinical protocols is essential to fully realize the potential of the ROX coupler device in hypertension management.

Carotid Body (CB) Ablation

Mechanism of action: The CB, situated at the carotid bifurcation, is a crucial peripheral chemoreceptor in humans. It plays a significant role in regulating respiratory and cardiovascular responses to changes in blood oxygen, carbon dioxide, and pH levels. The carotid sinus nerve, which innervates the CB, is involved in these regulatory processes. Animal experimental trials have demonstrated that carotid sinus nerve denervation can lead to reduced blood pressure (BP) levels [49].

Building on this concept, the Cibiem transvenous ultrasound system employs a minimally invasive approach to modulate CB activity [50]. This system uses ultrasound energy to target the CB, aiming to disrupt its function and consequently lower BP. The possible mechanism involves using targeted ultrasound waves to invoke CB disruption, which in turn leads to reduced chemoreceptor activity and decreased sympathetic nervous system. This subsequently leads to lowered BP.

Clinical trials and evidence: (1) Unilateral surgical CB resection. An early uncontrolled study assessed the effects of unilateral surgical CB resection in 15 patients with RH. The primary outcome was the change in the average office and ambulatory systolic BP (SBP) over a 12-month follow-up period [50]. While the overall results did not show a significant reduction in SBP, a subset of patients (eight out of 15) experienced notable reductions in daytime ambulatory SBP at three months (-23 ± 3 mmHg; p = 0.0005) and six months (-26 ± 4 mmHg; p = 0.0021), though this effect was not sustained at the 12-month follow-up [50].

Transvenous CB Ablation

More recent advancements have focused on less invasive techniques. A multicenter, first-in-man study evaluated the efficacy of transvenous CB ablation in patients with RH. This method uses catheter-based ultrasound to ablate the CB. The study reported a significant reduction in 24-hour ambulatory BP (ABP), with an average decrease of 9.1/6.7 ± 13.5/8.7 mmHg [51]. These findings suggest that this less invasive approach might offer a promising alternative for BP management in RH patients.

Challenges to implementation:

(1) Variability in response. The variability in patient response to CB modulation poses a significant challenge. While some patients experience substantial BP reductions, others do not show significant improvements. Understanding the factors contributing to this variability is crucial for patient selection and optimizing treatment outcomes.

(2) Long-term efficacy and safety. The long-term efficacy and safety of CB modulation need further investigation. The initial promising results must be validated through extended follow-up studies to ensure sustained BP control and identify potential long-term adverse effects.

(3) Technical and procedural challenges. The technical complexity of the procedure, especially for transvenous CB ablation, requires specialized skills and training. Ensuring that healthcare providers are proficient in these techniques minimizes procedural risks and improves patient outcomes. Challenges such as anatomical variability, risk of vascular damage, nerve injury, and thermal injury could limit its use. 

(4) Patient selection. Identifying the appropriate candidates for CB modulation is critical. Not all patients with RH may benefit from this intervention, and precise criteria need to be developed to ensure that only those most likely to respond are selected for the procedure. For example, it is probably not safe for patients with severe carotid stenosis or recent cerebrovascular diseases. It is also not advisable for patients with recent head and neck injuries/surgery.

(5) Cost and resource allocation. The cost of developing, implementing, and maintaining the necessary technology for CB modulation can be substantial. Ensuring these procedures are cost-effective and accessible to a broad patient population is essential for widespread adoption.

(6) Regulatory and ethical considerations. Regulatory approval processes and ethical considerations surrounding novel medical interventions must be thoroughly addressed including obtaining FDA approval while putting into consideration the potential benefits versus the risks of implementation. Ensuring that all trials are conducted with rigorous oversight and that patient safety is prioritized will be key to gaining regulatory approval and public trust.

CB modulation represents a novel approach to managing resistant hypertension, leveraging the body's own regulatory mechanisms to achieve BP control. Despite the promising early results from clinical trials, several challenges must be addressed to ensure its successful implementation. These include variability in patient response, the need for long-term efficacy data, technical complexities, patient selection criteria, cost considerations, and regulatory hurdles. Addressing these challenges through continued research, training, and careful clinical implementation will be crucial for realizing the full potential of this innovative treatment.

Pacemaker-Based Cardiac Neuromodulation

Pacemaker-based cardiac neuromodulation has emerged as a promising approach for managing hypertension, particularly in patients requiring pacemaker implantation [52]. This innovative technique leverages the capabilities of pacemaker technology to modulate cardiac function and subsequently reduce BP.

Mechanism of action: Pacemaker-based cardiac neuromodulation aims to reduce BP by modulating left ventricular ejection volume [52]. This is achieved through repeated adjustments of the atrioventricular intervals. The Moderato system, a key player in this approach, mimics a dual-chamber structure to optimize heart rhythm and reduce BP [52]. Altering the timing of ventricular contractions can influence cardiac output and vascular resistance, subsequently leading to a reduction in BP.

Clinical trials and evidence: (1) MODERATO-1 study. The MODERATO-1 study was a pivotal trial that assessed the efficacy of the BackBeat Moderato system. The Moderato-HTN study was an open-label, single-arm, multi-center, prospective trial. This study enrolled 35 patients with systolic BP exceeding 140 mmHg despite the use of antihypertensive medications [53]. Upon device activation, patients experienced a significant and immediate drop in BP, with a reduction of 24 mmHg in office systolic BP at the three-month follow-up mark [53]. This substantial decrease highlights the potential of the Moderato system to manage hypertension in pacemaker patients effectively.

(2) MODERATO-II studies. The subsequent MODERATO-II studies further supported the findings of the initial study. The MODERATO II Study was a prospective, multi-center, double-blind pilot study. These studies revealed a noteworthy drop of 11 mmHg in systolic BP among patients using the BackBeat Moderato system without significant adverse outcomes [54]. This evidence underscores the system's efficacy and safety in managing hypertension in patients requiring pacemakers.

Advantages and applicability: One of the most significant advantages of pacemaker-based cardiac neuromodulation is its applicability to patients who already need a pacemaker serving as a dual function. The Moderato system offers a tailored approach to BP control for these patients, integrating seamlessly with existing cardiac management strategies. This dual benefit of addressing both pacing needs and hypertension makes it an attractive option for a specific patient population.

Challenges to implementation: The primary challenge of this approach is its limited applicability. Pacemaker-based cardiac neuromodulation is inherently restricted to patients who require pacemaker insertion such as patients with arrhythmias such as sick sinus syndrome (SSS) or atrial fibrillation. This limits the potential patient population and may not be suitable for those with hypertension but without an indication of a pacemaker [55].

Adverse effects and safety concerns: While the MODERATO studies reported minimal significant adverse outcomes, the potential for adverse effects remains a concern most likely related to the device and procedure. Concerns such as vascular complications leading to bleeding/hematoma, device migration, thrombosis from foreign device in the artery, and infection were potential concerns. Additionally, excessive lowering of blood can lead to hypotension and even orthostatic hypotension. Adjusting the AV intervals to modulate BP could theoretically exacerbate conditions such as heart failure in susceptible patients. Continuous monitoring and robust safety protocols are essential to mitigate these risks.

Cost and resource allocation: The implementation of pacemaker-based cardiac neuromodulation involves considerable costs, both for the technology and the surgical procedures required for pacemaker implantation. This could burden healthcare systems and patients financially, potentially limiting widespread adoption.

Need for specialized training: Healthcare providers need specialized training to implement and manage pacemaker-based cardiac neuromodulation effectively. This includes understanding the intricacies of the Moderato system and being adept at adjusting AV intervals to optimize BP control without compromising cardiac function.

Long-term efficacy and research: Long-term efficacy and safety data are still required to establish the benefits and risks of pacemaker-based cardiac neuromodulation fully. Ongoing research and extended follow-up studies are necessary to validate the initial positive outcomes and ensure sustained BP control over time.

Pacemaker-based cardiac neuromodulation represents an innovative approach to hypertension management, particularly suited for patients requiring pacemaker implantation. The BackBeat Moderato system has shown significant potential in reducing BP, as evidenced by the MODERATO-1 and MODERATO-II studies. However, the implementation of this technology faces challenges, including limited applicability, potential adverse effects, high costs, the need for specialized training, and the necessity for long-term efficacy data. Addressing these challenges through continued research, training, and cost-management strategies will be crucial for maximizing the benefits of this promising approach to hypertension control.

Electro-Acupuncture

Electro-acupuncture, although initially developed for treating peripheral pain syndrome, has unexpectedly demonstrated efficacy in reducing BP. This technique involves the stimulation of the median nerve, which is thought to modulate the sympathetic nervous system, thereby influencing cardiovascular function and leading to lowered BP levels.

Clinical trials and evidence: Electro-acupuncture has been the subject of various clinical trials to evaluate its effectiveness in BP management. One notable study involved using the eCoin device, a minimally invasive electro-acupuncture device that delivers low-frequency electrical stimulation. This device is designed to provide 30 minutes of stimulation weekly, targeting the median nerve bilaterally.

Key findings: Significant reductions in BP were observed in a recent multi-center, prospective, double-blinded, sham-controlled, and randomized (1:1) trial utilizing the eCoin device. The study reported a mean BP reduction of over 10 mmHg after six months of treatment [56]. This result highlights the potential of electro-acupuncture as a viable non-pharmacological intervention for hypertension management [56].

Regulatory and approval status: Despite funding challenges during its development and trial phases, the eCoin device demonstrated sufficient efficacy and safety to gain regulatory approval. In March 2022, the device received approval from the US FDA for treating urinary urge incontinence, further validating its therapeutic potential [57]. It has yet to however receive FDA approval for Hypertension treatment.

Broader implications and potential: The successful application of electro-acupuncture for BP reduction has broader implications for its use in various medical conditions. Its mechanism of action through sympathetic nervous system modulation opens avenues for exploring its benefits in other autonomic dysfunction-related disorders. Furthermore, its minimally invasive nature makes it an attractive option for patients seeking alternatives to traditional pharmacological treatments compared to the previously discussed devices.

Challenges to implementation: Funding and resource allocation is a major challenge to implementing electro-acupuncture in controlling RH. The development and widespread adoption of electro-acupuncture devices such as eCoin require substantial financial investment. Initial trials faced significant funding challenges, which could impede further research and development efforts.

Another potential challenge is the standardization and protocol development. There is a need for standardized treatment protocols to ensure consistent and reproducible results across different clinical settings. Variability in stimulation parameters and techniques can affect the outcomes and efficacy of the treatment.

Healthcare provider training is another challenge, as effective implementation of electro-acupuncture in clinical practice necessitates comprehensive training programs for healthcare providers. Ensuring practitioners correctly apply the technique is crucial for its success.

Patient acceptance of electro-acupuncture as a treatment modality can vary. Factors such as the invasiveness of the procedure, cultural perceptions of acupuncture, and the need for regular treatment sessions may influence patient compliance.

Long-term efficacy and safety remain a concern. This is because, while short-term results are promising, electro-acupuncture's long-term efficacy and safety for BP management require further investigation. Longitudinal studies are needed to assess the sustainability of BP reductions and monitor for potential adverse effects over extended periods.

Incorporating electro-acupuncture into existing healthcare systems poses logistical challenges. Ensuring seamless integration with current hypertension management protocols and facilitating reimbursement through insurance systems are essential for widespread adoption. Electro-acupuncture represents a promising alternative for BP management, leveraging the modulation of the sympathetic nervous system through median nerve stimulation [56]. The clinical evidence, particularly from eCoin device studies, underscores its potential benefits. However, addressing the challenges related to funding, standardization, training, patient acceptance, long-term efficacy, and healthcare integration is crucial for realizing its full potential and ensuring its successful implementation in clinical practice.

Deep Brain Stimulation (DBS)

First introduced by Green Alexander Laurence in 2007, DBS emerged as a promising technique for reducing BP through targeted central nervous system stimulation. Initial studies revealed a substantial BP reduction of 25/8.4 mmHg when employing stimulation parameters set at 2 V and 30 Hz [58]. Subsequent cases further validated its efficacy; notably, a patient with chronic pain syndrome experienced a BP reduction of 33/13 mmHg following 27 months of sustained stimulation [59].

DBS involves the implantation of electrodes within specific brain regions, where electrical impulses are used to modulate neural activity. This technique has been widely adopted for managing neurological disorders such as Parkinson's disease, essential tremors, and dystonia [58]. Its mechanism of action in BP reduction is hypothesized to involve the modulation of autonomic pathways that regulate cardiovascular function.

Despite its therapeutic potential, several challenges impede the widespread adoption of DBS for hypertension management: The most important limiting factor for DBS is the high cost. The procedure is expensive, involving surgery, the implantation of the device, and ongoing maintenance and system adjustments. These financial barriers make it less accessible for many patients and healthcare systems [60].

Additionally, a potential limitation is its surgical risks: As with any invasive surgical procedure, DBS carries risks such as infection, hemorrhage, and adverse reactions to anesthesia. These risks necessitate a careful patient selection process and comprehensive preoperative evaluation.

Its technical complexity is also a potential challenge to the implementation of DBS. DBS's success requires precise electrode placement, which demands advanced imaging techniques and specialized surgical expertise. The complexity of the procedure can limit its availability to centers with highly skilled neurosurgical teams.

DBS also requires postoperative management, including regular follow-ups for device adjustments and monitoring for potential complications. This need for continual care can burden patients and healthcare providers.

There is also variable efficacy. While initial results are promising, the long-term efficacy of DBS for BP management remains under investigation. Variability in patient responses necessitates further research to identify success predictors and optimize stimulation parameters.

Additionally, the implantation of a brain device raises ethical questions and concerns about the psychological impact on patients. Issues such as consent, autonomy, and the potential for personality or cognitive function changes must be carefully considered.

While DBS offers a novel and potentially practical approach to hypertension management, significant challenges related to cost, surgical risks, technical complexity, and long-term efficacy must be addressed. Ongoing research and technological advancements are essential to overcoming these barriers and expanding the use of DBS in clinical practice.

Table 1 summarizes the available device therapies and their FDA approval status [61] as of November 2023.

**Table 1:** Different forms of device therapies for resistant hypertension (RH), mechanisms of action, FDA approval status, and limitations to implementation Table credits: Tolu-Akinnawo, Oluwaremilekun (one of the authors)

Therapy	Mechanism of action	FDA Approval status	Limitations
Renal Denervation (RDN)	This uses device-based technology to disrupt renal nerves, reducing sympathetic nerve activity and BP.	Clinically approved, however not FDA-approved for hypertension yet	Studies have shown varying efficacy. Procedural risks and costs are also potential barriers to implementation.
Baroreflex Amplification	Stimulates baroreceptors to reduce sympathetic outflow, heart rate, and peripheral resistance.	Not currently FDA-approved	The paucity of long-term data. Also, concerns about procedural risks and potential device complications are potential challenges to the implementation.
Arteriovenous Malformation	Creates an arteriovenous anastomosis to reduce systemic vascular resistance and BP.	Not currently FDA-approved	The invasiveness of the procedure and potential complications from arteriovenous shunt are common barriers to implementation. Limited data on efficacy and safety is also a potential barrier.
Carotid Body (CB) Ablation	Modulates carotid body activity via surgical resection or ultrasound modulation to reduce BP.	Not currently FDA-approved	Varying results in efficacy, potential procedural risks, and limited long-term studies are potential barriers to implementation.
Pacemaker-Based Cardiac Neuromodulation	Modulates left ventricular ejection volume through pacemakers to control BP.	Not currently FDA-approved for hypertension	Potential limitations to implementation are device implantation risks, potential for pacemaker-related complications, and limited data.
Electro-acupuncture	It uses low-frequency median nerve stimulation to reduce BP.	Not currently FDA-approved	Regular sessions are required, which could affect compliance. Also, varying efficacy and limited large-scale studies are potential barriers to implementation.
Deep Brain Stimulation	It involves central nervous system stimulation to reduce BP.	Not currently FDA-approved	The invasiveness of the procedure, the high cost, the potential for neurological side effects, and the limited number of studies are potential limitations and barriers to implementation.

Future directions

In the landscape of managing resistant and refractory hypertension, the future unfolds with promising avenues based on the insights gained from this comprehensive review of device-based therapies. As we navigate the evolving realm of cardiovascular health, several key directions emerge. A key area of continued research is the precision refinement of RDN. Further research is imperative to refine and personalize catheter-based RDN. Investigating advanced technologies, procedural techniques, and patient selection criteria will optimize the efficacy of this widely utilized therapy for resistant hypertension. Emphasis should be placed on longitudinal studies to assess its impact on cardiovascular outcomes and ascertain whether it confers benefits akin to pharmacological treatment. Additionally, exploring innovations in baroreflex amplification is imperative, as this therapy holds promise as a less invasive alternative, making it more appealing. The ongoing trials with devices such as the EVBA MobiusHD present opportunities for innovation. Future research should delve into the development of less invasive devices, assess long-term ambulatory blood pressure effects, and conduct rigorous randomized controlled clinical trials to confirm efficacy.

Additional efforts should also be directed towards advancements in arteriovenous malformation interventions. The ROX coupler device shows potential in addressing resistant hypertension through arteriovenous malformation. Future research should focus on refining the device design, evaluating its safety profile, and exploring its application in broader patient populations. Additionally, efforts should be directed toward mitigating potential risks associated with iliac venous stents. Continued research should also focus on unraveling the potential of CB ablation device therapy. Studies should aim to understand its sustained efficacy, safety profile, and potential impact on office and ambulatory blood pressure. More extensive randomized controlled trials are needed to ascertain its role in managing resistant hypertension. Additionally, the potential of pacemaker-based cardiac neuromodulation for hypertensive control, especially in pacemaker patients, warrants continued exploration. Future studies should delve into its applicability, long-term effects including tissue scarring/device problems/heart problems, and adverse outcomes, considering its viability as an option for those with resistant hypertension requiring pacemaker insertion.

Continued research is needed to validate the efficacy of electro-acupuncture device therapy. Although electro-acupuncture has shown promising BP reduction effects, it requires further validation through well-designed trials. Further research should aim to assess its long-term impact, scalability, and integration into routine clinical practice. Overcoming funding challenges is crucial to unlocking its potential as a minimally invasive strategy for hypertension management. Another focus of additional research is the evaluation of the feasibility and cost-effective Implementation of deep brain stimulation. While deep brain stimulation has shown significant BP reduction in various conditions, its adoption for hypertension management faces cost-related challenges, especially in low-resource settings. Future efforts should focus on optimizing the cost-effectiveness of this intervention, exploring its utility in specific patient subsets, and addressing barriers to broader implementation.

As we embark on these future directions, we must promote collaborative efforts among researchers, clinicians, and policymakers, who are key public health figures to translate these insights into tangible advancements, including more extensive research and clinical trials, ultimately enhancing the landscape of device-based therapies for resistant hypertension.

## Conclusions

In conclusion, the worsening trend of hypertensive disease and its complications continues to pose significant global public health concerns, necessitating continual advancements in treatment strategies. Device-based therapies, such as catheter-based RDN, baroreflex amplification, arteriovenous malformation, CB ablation, pacemaker-based cardiac neuromodulation, electro-acupuncture, and DBS, offer promising solutions for managing resistant hypertension. The evolving landscape, marked by FDA-approved US and RF RDN, underscores the significance of innovative interventions. Although several challenges continue to hinder their implementation, ongoing research emphasizes safety, efficacy, and long-term outcomes. As we navigate this complex terrain, these therapies showcase the potential to revolutionize hypertension management, providing an increased hope for improved patient outcomes and addressing the critical need for effective solutions in the face of resistant hypertension.
